# Porcine Circovirus Types 2 and 3: Detection and Association with the Productive Performance of Pigs on Commercial Farms in Southern Brazil

**DOI:** 10.3390/ani16213526

**Published:** 2026-11-07

**Authors:** Lucas Michel Wolf, Dalila Mabel Schmidt Tomm, Nicole Amoêdo Luvison, Rafael Sartori Flores, André Salvador Kazantzi Fonseca, Nilo Ikuta, Diéssy Kipper, André Felipe Streck, Vagner Ricardo Lunge

**Affiliations:** 1 Institute of Biotechnology, University of Caxias do Sul (UCS), Caxias do Sul95070-560, RS, Brazil; lmwolf@ucs.br (L.M.W.); naluvison@ucs.br (N.A.L.); rsflores2@ucs.br (R.S.F.); afstreck@ucs.br (A.F.S.); 2 Seara Alimentos LTDA, Caxias do Sul 95070-560, RS, Brazil; dalilatomm@gmail.com; 3 Simbios Biotecnologia, Cachoeirinha 94940-030, RS, Brazil; fonseca@simbios.com.br (A.S.K.F.); ikuta@simbios.com.br (N.I.); kipper.simbios@gmail.com (D.K.)

**Keywords:** porcine circovirus disease, PCV2, PCV3, qPCR, swine production

## Abstract

Porcine circoviruses 2 (PCV2) and 3 (PCV3) are endemic pathogens in swine populations worldwide. This study aimed to detect PCV2, PCV3, and genotype PCV2, and associate these PCV infections with pig productive performance on commercial Brazilian farms. Palatine tonsils were collected from 250 slaughtered pigs, originating from 25 batches/herds from 25 different farms (10 samples per batch/herd), and were used to identify PCV2 and PCV3. Overall, 23 (92%) herds tested positive for PCV2 and 19 (76%) for PCV3, with 18 (72%) herds positive for both these PCVs. Within-herd prevalence in the positive batches ranged from 10% to 100% for PCV2 and 10% to 50% for PCV3. The PCV2d genotype was identified in all positive herds, whereas PCV2b was only co-detected with PCV2d in five herds. Amino acid sequence analysis additionally revealed three main substitutions (A133H, T134D and L136M) in the Cap protein of most PCV2d samples evaluated here. PCV2 positivity in the herds correlated with mortality rate (ρ = 0.70; *p* = 0.001) and carcass condemnation (ρ = 0.57; *p* = 0.003), but not with average slaughter weight (ρ = −0.33; *p* = 0.102). PCV3 showed no significant correlation with pig productive performance (*p* > 0.05). In conclusion, PCV2 and PCV3 are widely and concurrently circulating on commercial pig farms in Southern Brazil, with PCV2d being the predominant genotype. The high frequency of PCV2 was associated with field mortality and carcass condemnation, highlighting the need for continuous molecular surveillance, prevention, and control of this circovirus in commercial swine production.

## Simple Summary

Pig farming is an important sector of global livestock production, with Brazil ranking among the world’s largest producers and exporters of pork. Maintaining the health of pig herds is essential to sustain productivity on farms. Porcine circovirus type 2 (PCV2) causes significant economic losses worldwide, resulting in mortality, impaired growth, and carcass condemnation at slaughter. Concurrently, porcine circovirus type 3 (PCV3) has emerged as a potential pathogen in commercial pig farms. Assessing the epidemiological dynamics of PCV2 and PCV3 under field conditions is crucial for defining prevention and control strategies for diseases associated with these pathogens in swine production. This study detected these two circoviruses and analyzed the productive performance in finishing pig herds on commercial farms in Southern Brazil. The results revealed that both viruses are widely circulating on the pig farms. In addition, PCV2—particularly the PCV2d genotype—was demonstrated to be the most concerning pathogen, since it was associated with higher mortality rates on farms and condemnation of carcasses in slaughterhouses. The control of these pathogens is essential to ensure the economic sustainability of swine production.

## 1. Introduction

Porcine circoviruses are a major concern in commercial swine health, representing an economic challenge for the pig industry worldwide [1]. Porcine circovirus type 2 (PCV2) has been associated with multisystemic clinical and subclinical manifestations, such as Post-weaning Multisystemic Wasting Syndrome (PMWS), characterized by progressive body condition decline and higher mortality rates in nursery and finishing phases, and Porcine Dermatitis and Nephropathy Syndrome (PDNS), a type III hypersensitivity reaction resulting in hemorrhagic skin lesions and severe renal failure. Porcine circovirus type 3 (PCV3) infection is similarly linked to PDNS, reproductive failure in sows (such as abortions and stillbirths), myocarditis in piglets, and systemic wasting. These clinical conditions normally result in high morbidity and mortality rates in the field, impaired average daily weight gain, herd/batch heterogeneity, and increased carcass condemnation rates at slaughterhouses [1,2,3,4].

PCV2 has been detected and identified in commercial pig farms since the late 1990s, while PCV3 has only been identified more recently [1]. Taxonomically, both viruses belong to the family *Circoviridae* and the genus *Circovirus*, officially classified as PCV2 and PCV3 [5]. This viral family also includes two other species: *Circovirus suis 1* (PCV1), a well-established non-pathogenic virus, and *Circovirus suis 4* (PCV4), whose pathogenicity, clinical relevance, and epidemiological importance in commercial swine herds remain under investigation [6,7]. As a result of dissemination and evolution, PCV2 and PCV3 have high genetic diversity. Currently, PCV2 is classified into nine genotypes (PCV2a to PCV2i), while PCV3 is classified into only three (PCV3a to PCV3c) [8,9].

PCV2 evolution has been historically characterized by genotype shifts in commercial pigs, starting with the initial dominance of PCV2a in the 1990s. In this early period, PCV2 caused severe clinical outbreaks of Post-weaning Multisystemic Wasting Syndrome (PMWS) with high mortality rates worldwide. This critical crisis prompted the widespread implementation of strict biosecurity measures and mass vaccination programs. However, immune and environmental selection pressures contributed to successive viral adaptations, driving the emergence and global transition to PCV2b in the 2000s and the subsequent emergence of PCV2d as the predominant circulating genotype since 2010 [1,10]. The remaining genotypes (PCV2c, PCV2e, PCV2f, PCV2g, PCV2h, and PCV2i) have only been sporadically detected in swine farms [1].

PCV3 emerged globally following its initial identification in the United States in 2016 [11]. Since then, it has been widely detected in commercial operations across the Americas, Asia, and Europe [12]. Currently, PCV3 is recognized as a ubiquitous and endemic agent in major swine-producing regions, circulating both in mono- and co-infection with PCV2 [1]. More definitive taxonomic and genotyping criteria for PCV3 are still under debate in the literature, with phylogenetic studies demonstrating different clades worldwide [13,14].

In Brazil, these two PCVs are endemic, presenting a wide geographical distribution. PCV2 has been reported since its global emergence in the 1990s, with severe clinical outbreaks and high mortality. In addition, PCV2 genotype epidemiological dynamics have already been reported in the country, with an initial high prevalence of PCV2a, a subsequent increase in the frequency of PCV2b in the 2000s, and the establishment of PCV2d as the most prevalent genotype from the 2010s onwards [1,15,16]. PCV3 has been only documented in recent studies, demonstrating co-circulation with PCV2 and occurrence across major swine-producing regions in the country [16,17,18,19].

The control and prevention of PCVs rely on biosecurity measures and immunization protocols in commercial farms. Biosecurity strategies encompassing all-in/all-out systems, strict cleaning, disinfection, and sanitary downtime protocols for facilities, alongside tight control over the movement of people and vehicles, and the provision of filtered water and air, are essential to minimize viral load in production environments [1]. Additionally, immunization with commercial vaccines is necessary for reducing viremia in pigs and improving productive performance [1,20]. Most current commercial vaccines are based on the PCV2a genotype, but there are growing concerns regarding effective cross-protection against circulating heterologous genotypes, particularly PCV2b and PCV2d. In contrast, no commercial vaccines are currently available on the market for PCV3 [1,3,20].

PCV2 and PCV3 must be monitored in production farms, including characterization of the main genotypes, to guide regional biosecurity strategies and define immunization programs [1,21]. The objective of this study was to detect PCV2 and PCV3 in pigs from commercial farms sent to slaughter, as well as to evaluate the association between infection with these PCVs and pig productive performance in Brazilian swine herds.

## 2. Materials and Methods

### 2.1. Study Area and Sampling

The study was conducted using samples from pig carcasses collected by convenience at a federally inspected slaughterhouse located in the municipality of Caxias do Sul, Rio Grande do Sul (RS), Brazil. The samples were obtained from animals of the same genetic line (Agroceres PIC^®^—Landrace × Large White) originating from 25 herds across 25 different pig farms, which are situated in 23 distinct municipalities. The animals from each herd were sent for slaughter in batches (on different days for each batch) between November 2024 and October 2025 (Figure 1; Table 1).

The evaluated herds originated from high-tech commercial production systems operating under all-in/all-out management and strict biosecurity protocols in the farms. All animals were subjected to the standard vaccination program of the production system, receiving immunization against PCV2 and *Mycoplasma hyopneumoniae* at weaning.

Pig productive performance and health data of the 25 herds/batches were retrieved from the computerized management system of the officially inspected slaughter facility. For each sampled batch/herd, the following data were collected: slaughter date, mean slaughter age (days), mean slaughter weight (kg), total finishing mortality rate (%), and total carcass condemnation rate recorded at the slaughter line (%). The last three data were used as pig productive parameters for this study.

### 2.2. Sample Collection

A total of 250 palatine tonsils were collected from individual pig carcasses destined for slaughter, originating from 25 batches of 25 herds from different commercial farms (10 tonsils per batch). The 10 carcasses per batch were randomly sampled on spaced-out collection days, according to the operational feasibility of the slaughterhouse.

All samples were collected post-mortem in a routine sanitary inspection at an officially inspected industry, with no animals handled or slaughtered specifically for this study. Immediately following official inspection, each palatine tonsil was dissected using individual sterile scalpels or surgical forceps and scissors. Strict cross-contamination control measures were applied between individual animals: sampling instruments were thoroughly cleaned and disinfected with a sodium hypochlorite solution prior to each collection, and the operator replaced disposable gloves for every sampled carcass. Collected tonsil fragments were individually packaged in sterile containers, transported on ice to the laboratory, and stored at −80 °C until molecular processing.

### 2.3. DNA Extraction

Palatine tonsil samples were subjected to mechanical processing to obtain a homogeneous suspension. Initially, tissue fragments of approximately 1 mm were sectioned using sterile disposable scalpels. Subsequently, the tissue was transferred to porcelain mortars and ground with pestles, using 3 g of sterile sand as an abrasive agent and 1 mL of physiological saline solution (0.9% NaCl) as a diluent. The entire procedure was performed in a unidirectional laminar flow biosafety cabinet, adhering to strict aseptic protocols to prevent cross-contamination between samples. Homogenization was carried out until a visually homogeneous suspension free of macroscopic solid fragments was obtained. Following homogenization, the homogenate was transferred to Eppendorf tubes and centrifuged at 10.000× *g* for 5 min to sediment cellular debris and sand. The resulting supernatant was collected in aliquots and stored at −80 °C for subsequent genomic DNA extraction. DNA extraction was performed using 50 µL of the supernatant with the commercial Prep and PreAmp kits (NewGene, Simbios Biotecnologia, Cachoeirinha, RS, Brazil), following the manufacturer’s instructions.

### 2.4. Molecular Detection of PCV2 and PCV3

Detection of PCV2 and PCV3 DNA was performed using real-time semi-quantitative Polymerase Chain Reaction (qPCR). The reactions were performed using the commercial kits NewGene PCV2Amp (PCV2) and PCV3Amp (PCV3) following the manufacturer’s recommendations (Simbios Biotecnologia, Cachoeirinha, Brazil). All assays were carried out in a thermal cycler (QuantStudio 5, Thermo Scientific, Waltham, MA, USA) under the following conditions: an initial denaturation cycle at 95 °C for 3 min, followed by 40 cycles at 95 °C for 15 s and 60 °C for 60 s. Synthetic positive controls NewGene PCV2Ref and PCV3Ref (Simbios Biotecnologia, Cachoeirinha, RS, Brazil) were included in separate qPCR tubes in all assays.

PCV2 and PCV3 were evaluated according to cycle threshold (Ct) value determination. For this purpose, amplification curves were analyzed using the instrument’s software (QuantStudio Design and Analysis Software v.1.5), with baseline values automatically adjusted and the Ct threshold manually fixed within the exponential amplification phase for each virus. Samples presenting typical sigmoidal amplification curves and Ct values within the linearity range of the standard curve were classified as positive, whereas those with no detectable amplification were considered negative. Ct values were used as a semi-quantitative measure [11,21,22,23]. Samples with a Ct < 21 were classified as having active replication consistent with the development of PCVAD, whereas Ct values between 21 and 35 were interpreted as representing a moderate to low-level infection. Positive and negative control samples for PCV2 and PCV3 were also included in all assays.

### 2.5. PCV2 Genotyping and Phylogenetic Analysis

PCV2 genotyping was performed by sequencing the capsid protein-coding region (*cap* gene) of one to three samples per herd/batch, based on the availability of PCV2-positive samples with Ct values low enough to allow for the amplification of larger fragments for sequencing (Ct < 30). The target region was amplified using a PCR protocol previously described [24]. The amplified products were purified and subjected to bidirectional Sanger sequencing using the same PCR primers. Sequencing chromatograms were inspected for quality, and forward and reverse reads were assembled into consensus sequences using Geneious v.2021.2.2 (Biomatters Ltd., Auckland, New Zealand). PCV2 sequences generated in this study were deposited in GenBank under accession numbers QB037558-QB037605 (Table S1).

For phylogenetic analysis, the consensus sequences obtained in this study were aligned with representative reference sequences of nine PCV2 genotypes (PCV2a to PCV2i) retrieved from GenBank (Table S2), using MAFFT v.7 [25]. A maximum-likelihood (ML) phylogenetic tree was inferred using the W-IQ-TREE web server [26]. The best-fit nucleotide substitution model was selected with ModelFinder [27], and branch support was assessed using 1000 ultrafast bootstrap replicates [28]. Bootstrap values ≥70% were considered to indicate well-supported clades [29]. The final phylogenetic tree was visualized and edited using FigTree v.1.4.4.

### 2.6. Analysis of Amino Acid Substitutions

An amino acid alignment of the deduced capsid protein-coding region of PCV2 was generated using MAFFT v.7 [25]. The resulting alignment was imported into Geneious v.2021.2.2 (Biomatters Ltd., Auckland, New Zealand), where the main amino acid substitutions were visually inspected and evaluated.

### 2.7. Statistical Analysis

Data were analyzed using IBM SPSS Statistics (v.29.0). Initially, descriptive statistics were performed to determine the positivity frequency (percentage) for PCV2 and PCV3, as well as the prevalence of co-detection at the herd level (simultaneous detection of both viruses). To evaluate the association between viral positivity rates and herd-level performance indicators, commercial swine batches (n = 25, with 10 animals sampled per herd/batch) were defined as the experimental unit. PCV2 and PCV3 infection frequency were calculated as the percentage of qPCR-positive samples relative to the total analyzed within each herd/batch. Due to the sample size (n = 25) and non-parametric distribution of certain continuous performance variables, Spearman’s rank correlation coefficient (ρ) was applied to assess monotonic relationships between viral positivity rates and dependent productivity/health indicators: mean slaughter weight (kg), finishing phase mortality rate (%), and slaughterhouse carcass condemnation percentage (%). Shapiro–Wilk test was initially performed to assess data normality and guide the selection of non-parametric methods. Statistical significance was established at *p* < 0.05 for all analyses. Nine correlation tests were run (6 virus–outcome + 3 outcome–outcome) without correction for multiplicity.

## 3. Results

### 3.1. Detection of PCV2 and PCV3

PCV2 was detected in 23 of the 25 herds evaluated (92%), while PCV3 was present in 19 herds (76%), with co-circulation by both viruses identified in 18 herds (72%). In only two herds (8%), PCV2 was not detected in the 10 sample tonsils. Overall, there were 125 (50%) animals positive for PCV2 and 40 (16%) for PCV3. The percentage of positive animals varied by herd/batch, with PCV2 frequency ranging from 10% to 100% and PCV3 frequency from 10% to 50% (Table 1).

The Ct values for PCV2 and PCV3 varied considerably among the samples (17.9 to 38.8), with notably high quantities of PCV2 DNA (Ct < 21) observed in some animals (from herds CO: Ct 20.7, CR: Ct 18.5, GU: Ct 19.7, PL: Ct 20.5, and SN: Ct 17.9), possibly reflecting viral replication and tissue infection in these individuals in the respective herds (Table S3). Co-infection of PCV-2 and PCV-3 was identified in 7.6% of the animals (19/250).

### 3.2. PCV2 Phylogenetic Analysis and Genotyping

Regarding PCV2 genotyping, one sequence was obtained from each of three herds, two sequences from each of 15 herds, and three sequences from each of five herds (Table S1). Based on the ModelFinder Bayesian Information Criterion (BIC) implemented in IQ-TREE, the most suitable nucleotide substitution model was HKY+F+R2. The phylogenetic analysis included the 48 sequences of the PCV2 *cap* gene generated in this study (Table S1) and 20 reference sequences representing recognized PCV2 genotypes retrieved from GenBank (PCV2a, *n* = 3; PCV2b, *n* = 5; PCV2c, *n* = 1; PCV2d, *n* = 5; PCV2e, *n* = 1; PCV2f, *n* = 1; PCV2g, *n* = 1; PCV2h, *n* = 1, and PCV2i, *n* = 2). One PCV1 sequence (GenBank accession number AF071879) was included as an outgroup to root the tree (Table S2).

Phylogenetic reconstruction showed that six sequences generated in this study clustered within the PCV2b genotype, while 42 sequences clustered within the PCV2d genotype (Figure 2 and Figure S1). Both genotype-specific clades were strongly supported, with ultrafast bootstrap values of 100%.

Genotype analysis revealed that PCV2d was present in all 23 PCV2-positive herds (100%). In addition, the PCV2b genotype was detected exclusively in co-circulation with PCV2d in five (20%) of the PCV2-positive herds (Table 1). PCV2a was not identified in any of the sequenced samples.

### 3.3. Amino Acid Profile

The alignment of the amino acid (aa) sequences of the capsid protein (Cap/ORF2) revealed distinct patterns that differentiated the PCV2 genotypes in the herds/batches studied (Table 2). All sequences classified as PCV2b (*n* = 6) fully retained the previously described amino acid signature sequence of this genotype, exhibiting 57I, 80L, 86S, 88P, 89R, 91V, and 121S. However, five of them (83.3%) presented the T134P substitution. In addition, sequences assigned to the PCV2d genotype (*n* = 42) exhibited some hallmark residues, including 53I, 80L, 86S, 88P, 89L, 90T, and 91V. However, the more C-terminal motif (from aa 133 to 136) presented three important substitutions (A133H, T134D, and L136M) in the majority of the PCV2d sequenced here (Figure 3). Two other substitutions were further observed: R39K (*n* = 17) and K132R (*n* = 1). Notably, only a single PCV2d sequence maintained the typical 133–136 C-terminal extension motif.

### 3.4. Association with Pig Productive Parameters

Spearman’s correlation analysis between the positive sampling frequency and productive parameters revealed distinct patterns according to the occurrence and frequency of the two PCVs.

For PCV2, a moderate-to-strong, statistically significant positive correlation was observed with field mortality rate (ρ = 0.70; *p* = 0.001) and slaughterhouse carcass condemnation percentage (ρ = 0.57; *p* = 0.003) (Figure 4). The increased proportion of PCV2-infected animals within herds was associated with elevated losses during the finishing stage of production and higher carcass condemnations during sanitary inspection. Conversely, the association between PCV2 frequency and mean slaughter weight (ρ = −0.33; *p* = 0.102), although inversely proportional, was not statistically significant.

For PCV3, viral detection frequency showed no statistically significant correlation with any of the evaluated productive parameters (*p* > 0.05) (Figure 4). The coefficients obtained for mean slaughter weight (ρ = −0.19; *p* = 0.353), field mortality (ρ = 0.02; *p* = 0.938), and slaughterhouse condemnation rate (ρ = −0.33; *p* = 0.160) indicate that, in the sampled batches, the isolated presence of PCV3 had no measurable impact on herd zootechnical performance.

In assessing the interrelationships among herd zootechnical parameters, a moderate-to-strong, statistically significant negative correlation was observed between mean slaughter weight and slaughterhouse condemnation rate (ρ = −0.60; *p* = 0.001). This finding demonstrates that batches reaching lower mean weights at slaughter were associated with higher carcass condemnation percentages during sanitary inspection. Conversely, associations involving field mortality rate did not achieve statistical significance (*p* > 0.05), exhibiting a weak, inversely proportional trend with mean slaughter weight (ρ = −0.29; *p* = 0.156) and a weak, direct correlation with slaughterhouse condemnation rate (ρ = 0.22; *p* = 0.284) (Table 3). Furthermore, the relationship between the average daily weight gain per herd (ADWG) and the prevalence of PCVs was evaluated; no statistically significant correlations were observed for either PCV-2 (ρ = −0.329; *p* = 0.108) or PCV-3 (ρ = −0.093; *p* = 0.657) (Table S5).

## 4. Discussion

Brazilian swine production holds a prominent position in the global animal protein market, supported by a technified production chain with high health status [30,31]. However, intensive production and high population density favor the persistent circulation of endemic viral agents [20]. Among the major viral pathogens, PCVs (highlighting PV2 and PCV3) have been considered significant concerns due to their impact on health and the associated economic losses [1,12,32]. Previous studies have already demonstrated the wide dissemination of these two circoviruses in commercial herds across various producing regions of Brazil [16,17,18,19,33]. Therefore, the present study investigated the current occurrence of these concerning viral pathogens and also conducted a preliminary analysis of the potential zootechnical impact on swine herds in commercial farms in Southern Brazil.

First, the effective detection of these two circoviruses in the majority of animals from batches sent for slaughter at an abattoir—using easily collected tonsil samples and a robust qPCR methodology—validates an efficient and practical procedure for assessing pathogenic viruses in swine herds. As a key secondary lymphoid organ of the oropharyngeal cavity and a major entry point for various viral pathogens, the tonsils serve as sites of persistence and typically harbor high loads of these microorganisms, enabling pathogen detection in both clinically healthy and diseased animals [1,3]. The wide range of Ct values (17.9 to 38.8) observed in individual samples further allows us to estimate specific viral quantities for these PCVs in pigs, including potential associations with clinical manifestations. Thus, this study reinforces previous research conducted at slaughterhouses, which demonstrated the feasibility and sensitivity of monitoring tonsillar tissue for the comprehensive screening of subclinical PCV2 and PCV3 infections, as well as other pathogens, in livestock [34,35,36].

PCV2 was detected in 92.0% (23/25) of the herds and in 50% (125/250) of the animal individual carcasses, demonstrating the widespread dissemination of this highly pathogenic virus in pig finishing farms in Brazil, in agreement with previous national and international surveys [2,16,19,21,32]. In Europe, a previous study demonstrated individual positivity rates in finishing pigs ranging between 0% and 70% depending on the country [21]. In Brazil, studies using different biological samples from slaughter and production animals have reported PCV2 detection rates exceeding 90% of the herds and reaching 64% of the individual animals [19,32]. This striking concordance in herd-level frequencies reflects the endemic and sustained circulation of PCV2 in commercial farms, demonstrating that, even in the face of systematic vaccination, viral circulation remains widely distributed in the termination stage of production [2].

Furthermore, PCV2 load has been proposed as a determining factor for the onset of clinical disease, with Ct values lower than 21 in animal serum associated with characteristic lymphoid lesions [37]. In the present study, five animals showed Ct values below this cutoff (Ct < 21). Although a different tissue sample was used here (tonsils), these high viral quantities suggest intense tissue viral replication and a strong probability that the pigs could present clinical manifestations or more severe forms of subclinical infection [3,10]. Unfortunately, individual clinical assessments of these pigs were not performed, as the samples were obtained directly from carcasses at the slaughterhouse rather than from animals monitored in vivo on the farms [2,20].

PCV3 was also detected frequently in the same herds (76.0%; 19/25) and individual animal carcasses (16.0%; 40/250), supporting the endemic nature of this virus in Southern Brazil. Previous surveys in Brazil demonstrated frequencies around 30% [18,19,32]. This epidemiological profile reflects the cosmopolitan pattern of PCV3 also observed across the Americas, Asia, and Europe [11,12,13,14]. In international samplings of finishing and slaughter pigs, PCV3 detection rates fluctuate between 8% and 20% at the individual level. Here, the demonstrated prevalence aligns with the “clinically silent” behavior of this virus in global swine production [21,38].

Also noteworthy is the occurrence of PCV2 and PCV3 co-infection in 7.6% of the samples (19/250). The concomitant detection of both species in field sampling is widely reported in the literature and suggests that infection by one circovirus does not establish cross-immunity or prevent tissue infection by the other [19,21,22,32,39]. Notably, in co-infected cases, the mean PCV2 Ct dropped to 25.7, indicating a higher viral DNA load in animals infected by these both PCVs.

In addition, this study evaluated the association between the detection of PCV2 and PCV3 and mortality rates on the farm, as well as carcass condemnations at slaughter. Correlation analyses revealed distinct production patterns for these two viral species in the herds evaluated. For PCV2, a positive and statistically significant correlation was observed with finishing pig mortality rate (ρ = 0.70; *p* = 0.001) and the slaughterhouse carcass condemnation percentage (ρ = 0.57; *p* = 0.003). These results suggest that a higher prevalence of PCV2 in the herds may be associated with poorer health status, a higher mortality due to PCV2, and consequent production losses. PCV2 is the etiological agent of a complex spectrum of clinical and subclinical diseases that induce lymphoid depletion and immunosuppression, favoring co-infections with other pathogens of the porcine respiratory disease complex [8,32,33]. In herds facing persistent health challenges, the active circulation of PCV2 can negatively compromise zootechnical performance and the economic viability of the operation [2,10]. In contrast, the detection of PCV3 alone showed no statistically significant correlation with average slaughter weight, mortality rate, or carcass condemnations. Since PCV2 and PCV3 co-circulated in most herds, isolating the specific clinical impact of PCV3 remains a challenge under field conditions. In the only herd where PCV3 was detected in the absence of PCV-2 (“Sério”), no overt clinical disease or unusual mortality was reported, suggesting that, at most, infection with this virus may be associated with a subclinical condition, as previously demonstrated [1,14]. However, PCV3 has already been reported in association with systemic disorders, such as reproductive failure in sows, PDNS-like lesions, and myocarditis in piglets [11,18].

An additional analysis of productive indicators revealed a strong negative correlation between mean slaughter weight and carcass condemnation rate (ρ = −0.59; *p* = 0.001). Herds that reached lower slaughter weights exhibited higher condemnation percentages on the sanitary inspection line. This association reflects the impact of chronic infectious processes that reduce average daily weight gain and lead to tissue lesions detected during carcass inspection [8,20,32].

As PCV2 was associated with reduced pig productive performance in Brazil, additional analyses of the circulating genotypes were performed in all positive swine herds. It was clearly revealed that the PCV2d genotype accounted for 87.5% of the analyzed sequences (42/48), whereas PCV2b was identified in 12.5% (6/48). This high frequency reflects a well-documented global trend of the genotype shifting toward the predominance of PCV2d, which is consistent with recent data from other epidemiological surveys in Europe and Asia [21,40,41]. An additional comparison with reference sequences representing genotypes PCV2a–PCV2i described in previous studies provided a broader genetic context for the classification of the Brazilian strains and enabled their relationships with the recognized diversity of PCV2 genotypes to be assessed [42,43,44,45,46,47,48,49,50].

Furthermore, the amino acid sequence of the capsid protein (Cap/ORF2) was evaluated for all samples. Specific residue substitutions in PCV2b and PCV2d are well-established molecular markers for genotypic differentiation. As the primary target of the protective immune response and the site of neutralizing epitopes, the structural conservation of the Cap protein is crucial. The triple A133H, T134D, and L136M amino acid substitutions in most PCV2d sequenced here are located in the EF loop (residues 124–149), a surface-exposed region of the capsid that acts as a mutational hotspot and a critical antigenic site. Mutations in this loop and in surface-exposed regions arise from immune selection pressure and could induce antigenic drift. Consequently, there is also the possibility of reduced efficacy of the cross-immunity conferred by conventional commercial vaccines (based on PCV2a, PCV2b, or even the classic PCV2d strain) [40,41].

It is also important to highlight that the herds analyzed here were immunized against PCV2 at the time of weaning. However, vaccination alone may not completely prevent infection by field strains [1,20]. The detection of the virus in the lymphoid tissues of vaccinated animals—combined with the predominant occurrence of a distinct PCV2d strain (which even exhibits changes in the amino acid sequences of the Cap protein) and, to a lesser extent, PCV2b—suggests the likely widespread circulation of viral field variants. Although heterologous vaccines based on PCV2a are highly effective in preventing the acute clinical form of the disease, weaker cross-immunological protection against heterologous mutant genotypes facilitates the subclinical replication of other genotypes; this has, in fact, been previously demonstrated for PCV2d and PCV2b [1,16].

Although these data have epidemiological and clinical implications, they only represent preliminary evidence. It is important to highlight that some other common pathogens in Brazilian swine production (such as *Mycoplasma hyopneumoniae*, *Actinobacillus pleuropneumoniae*, *Glaesserella parasuis*, *Salmonella*, *Lawsonia*, etc.) were not analyzed in the herds of this study. Also, other factors that could interfere with the health and performance of the finishing herds (such as herd size, farm/integrator, vaccination and immunization time, season, flow conditions) were not obtained, limiting the scope of some findings. More robust epidemiological studies are needed to determine the true prevalence of these viruses (PCV2 and PCV3), as well as the specific PCV2 genotypes and their impact on the health of the Brazilian swine production chain. Further studies with larger sample sizes are also needed to achieve statistical significance regarding the causal clinical impact and the extent of performance losses caused by these PCVs in commercial farms.

In summary, the results here confirm the need for continuous molecular monitoring and characterization of circulating porcine circoviruses in Southern Brazil [16,18,19]. The high prevalence of PCV2d and its association with reduced zootechnical performance highlight the importance of improving internal biosecurity controls and management practices. Specifically, optimizing cleaning and disinfection protocols between herds, strictly implementing an “all-in/all-out” management system, and auditing PCV2 vaccination schedules contribute to robust herd immunity in the face of high viral pressure in the field. Furthermore, as there are currently no commercial vaccines available against PCV3, ongoing epidemiological surveillance of this circovirus is essential for maintaining sanitary standards and the productivity of the Brazilian swine industry [1,20].

## 5. Conclusions

This study detected PCV2 and PCV3 in commercial swine herds from Brazil. PCV2 infection rate was associated with increased finishing-stage mortality and carcass condemnations. Molecular analysis revealed a predominance of the PCV2d genotype contrasted with a minor frequency of PCV2b. In addition, analysis of the amino acid profile revealed key point mutations in the PCV2d sequences within the C-terminal motif ^133^HDAM^136^. These findings reinforce the importance of continuous epidemiological surveillance for enhancing sanitary control and immunological strategies in the swine industry in Brazil.

## Figures and Tables

**Figure 1:**
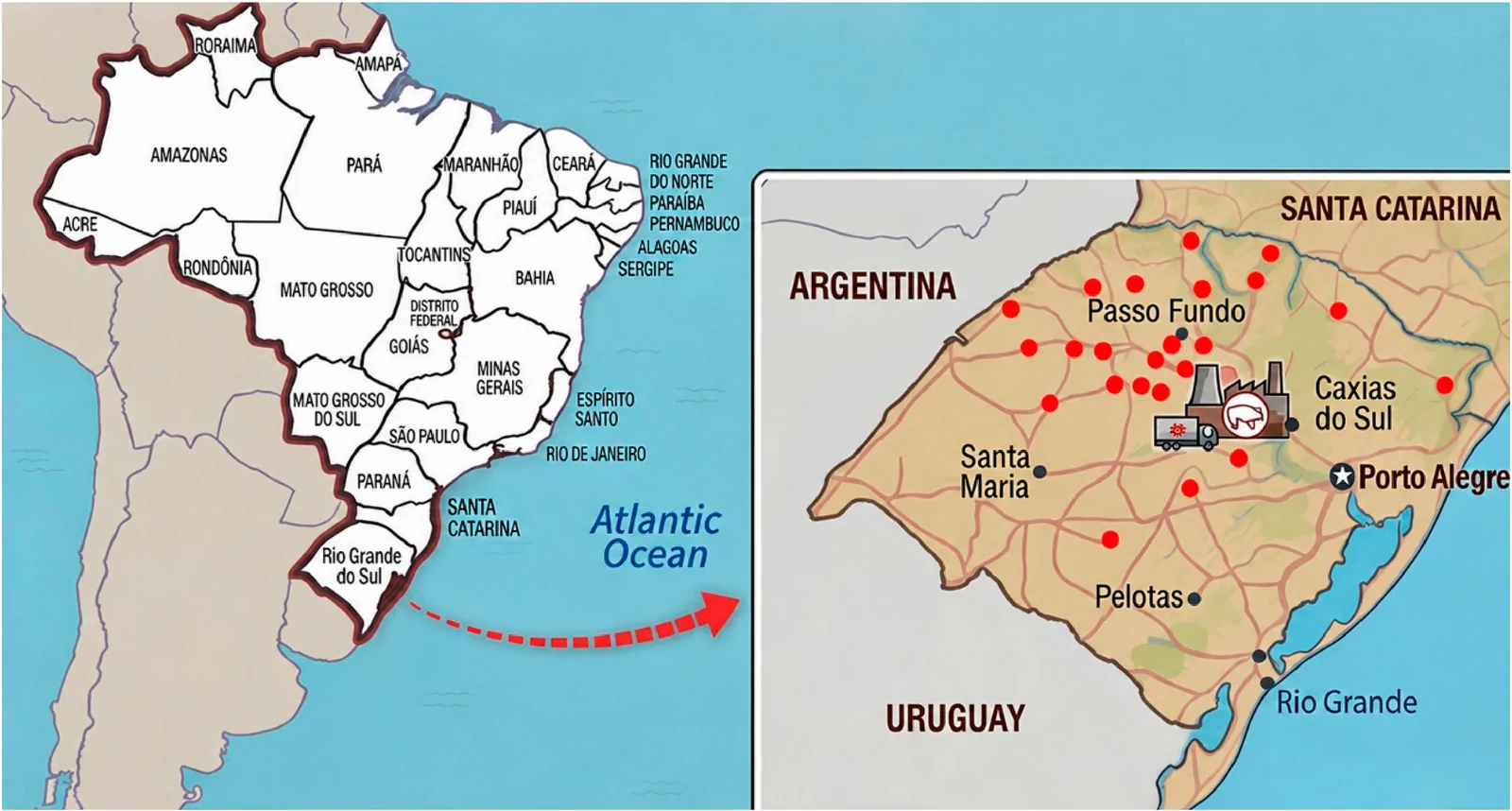
Geographic representation of the origin of pig herds/batches (red dots) and the swine slaughterhouse (factory icon) located in the municipality of Caxias do Sul (RS).

**Figure 2:**
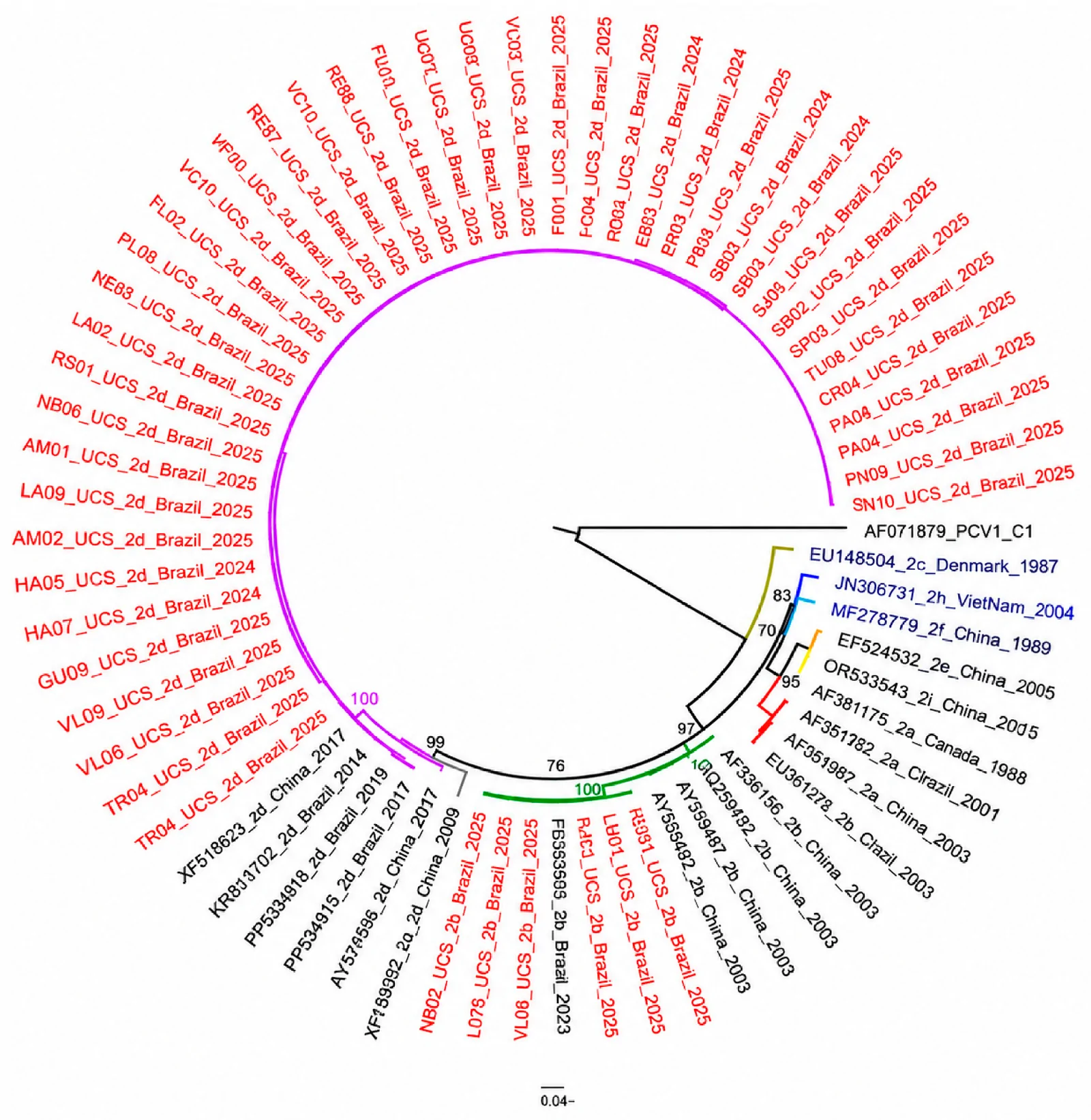
Maximum-likelihood phylogenetic tree based on the PCV2 *cap* gene nucleotide sequences. The analysis included 48 PCV2 sequences generated in this study, 20 reference sequences representing the recognized PCV2 genotypes (PCV2a to PCV2i) retrieved from GenBank, and one PCV1 sequence (GenBank accession number AF071879) used as the outgroup. Sequences generated in this study are highlighted in red. Clade colors indicate the corresponding PCV2 genotypes: PCV2a (red), PCV2b (green), PCV2c (brown), PCV2d (purple), PCV2e (orange), PCV2f (teal), PCV2g (gray), PCV2h (blue), and PCV2i (yellow). The tree was inferred using the maximum-likelihood method with 1000 ultrafast bootstrap replicates. Bootstrap support values are shown at the major nodes.

**Figure 3:**
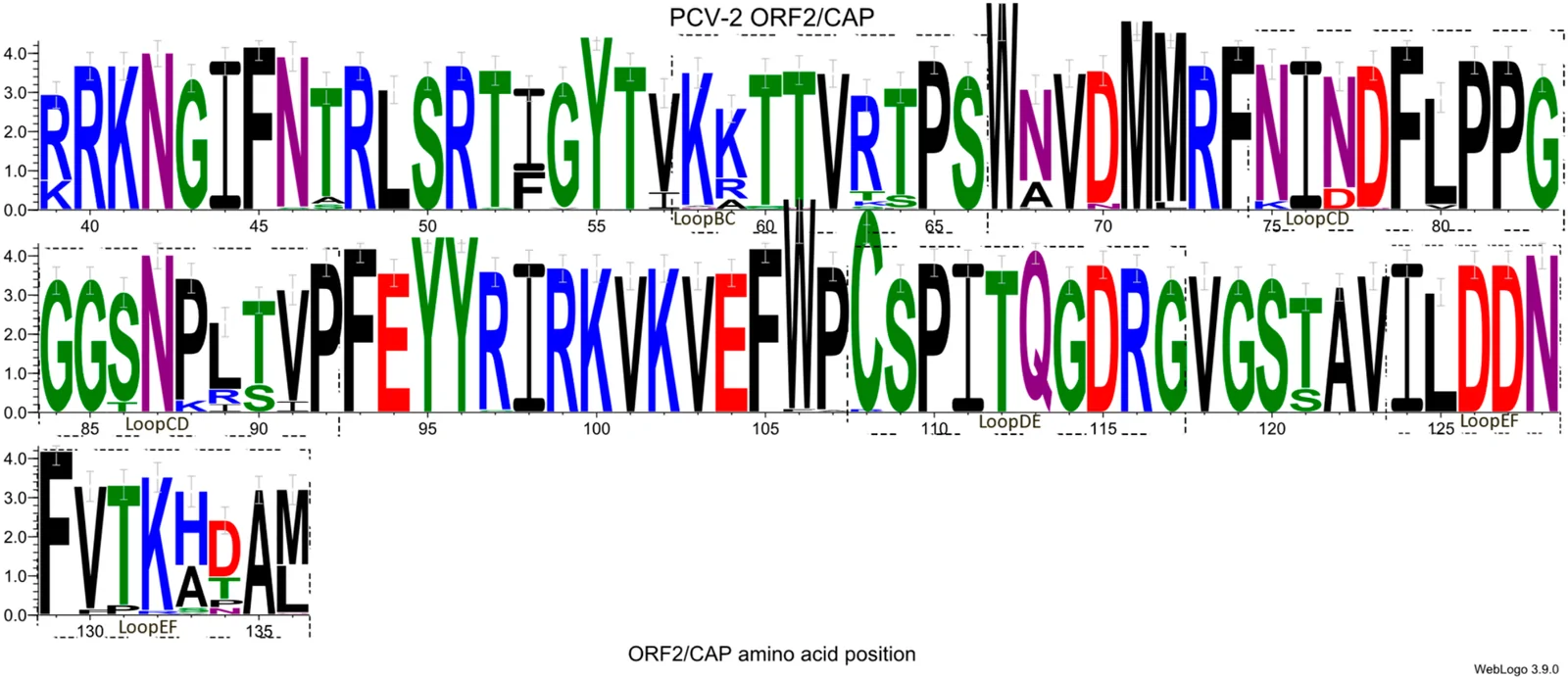
Sequence logo of the PCV2 capsid protein based on the amino acid sequence alignment of 20 reference sequences and 48 sequences generated in the present study. The complete amino acid sequence alignment is provided in Table S4. Each amino acid is represented by its single-letter abbreviation and colored according to its chemical properties, as defined in WebLogo 3: polar (green), neutral (purple), basic (blue), acidic (red), and hydrophobic (black). The x-axis indicates amino acid positions in the PCV2 capsid protein, while the y-axis represents information content in bits. The BC, CD, DE, and EF loop regions are indicated below the corresponding positions.

**Figure 4:**
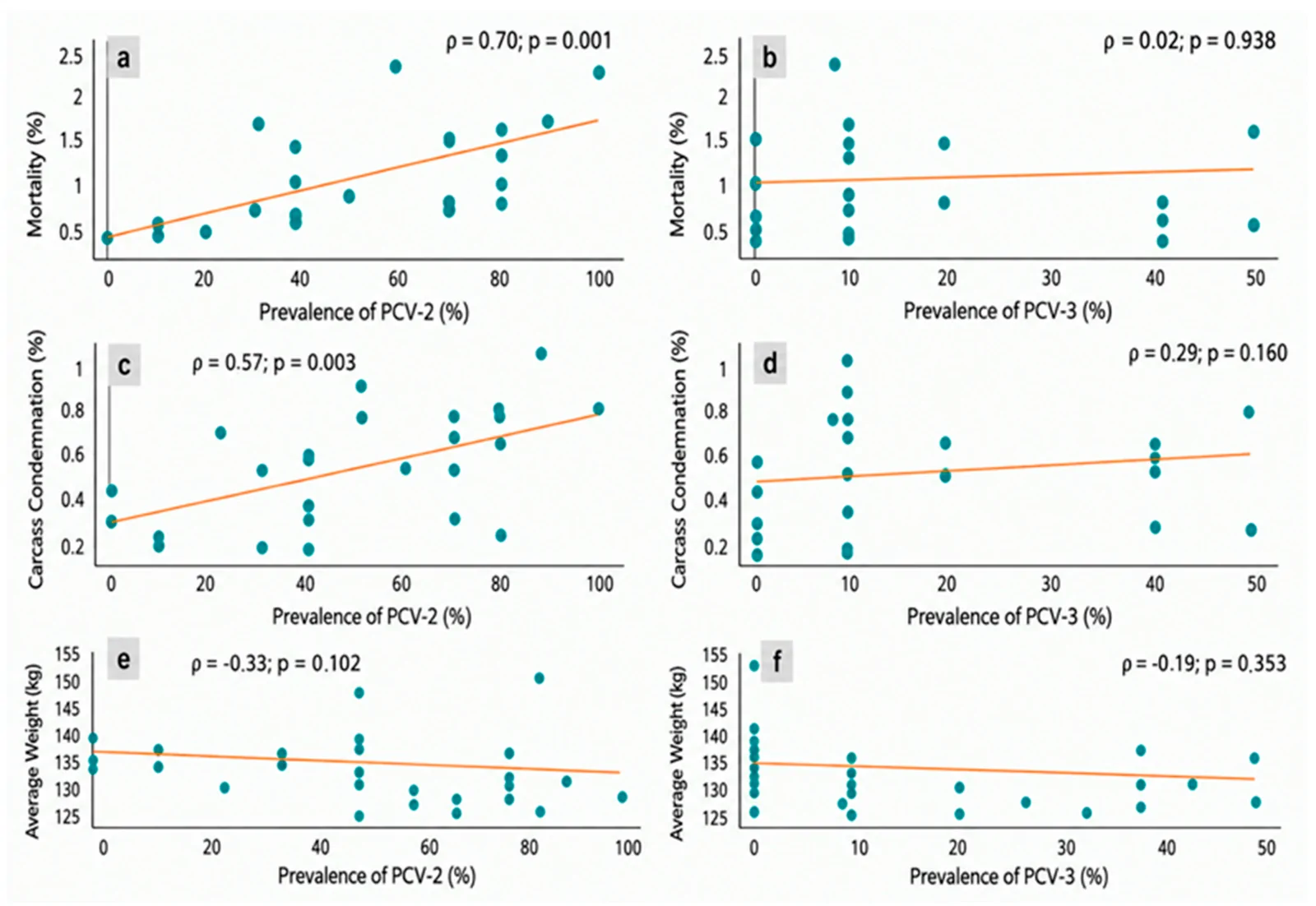
Correlation between PCV2 and PCV3 prevalence and zootechnical parameters. (**a**,**b**) Mortality rate during the finishing phase (%); (**c**,**d**) carcass condemnation rate at slaughter (%); and (**e**,**f**) average slaughter weight (kg), in relation to PCV2 and PCV3 prevalence, respectively. Correlation coefficients (ρ) and significance values (*p*) are indicated in the upper corner of each panel (*p* < 0.05 indicates a statistically significant association).

**Table 1:** Detection and frequency of PCV2/PCV3 and productive performance parameters of pigs in the 25 swine herds/batches.

Herd/ Batch	Municipality	PCV2 (*n*/%)	Genotype	PCV3 (*n*/%)	Slaughter Age (Days)	Average Weight (kg)	Mortality (%)	Condemnation (%)
1-AM	Arroio do Meio	4/40	2b + 2d	4/40	182	136	0.61	0.63
2-CO	Cotiporã	9/90	2d	1/10	184	128	1.68	1.07
3-CR	Crissiumal	8/80	2d	1/10	178	129	1.31	0.80
4-ER	Erechim	10/100	2d	1/10	189	125	2.37	0.81
5-FV	Fagundes Varela	8/80	2d	5/50	177	133	1.62	0.84
6-GU	Guabiju	5/50	2d	1/10	185	126	0.90	0.92
7-HA	Harmonia	4/40	2d	0/0	194	148	1.06	0.17
8-LA	Lajeado	4/40	2d	4/40	182	135	0.61	0.32
9-NB	Nova Bassano	7/70	2b + 2d	2/20	184	129	0.82	0.70
10-PA	Palmitinho	8/80	2d	4/40	179	124	0.80	0.70
11-PL	Porto Lucena	7/70	2b + 2d	1/10	183	127	0.74	0.81
12-RE	Relvado	6/60	2d	4/40	181	124	2.40	0.57
13-RS	Roca Sales	1/10	2d	1/10	180	131	0.45	0.20
14-SE	Sério	0/0	-	4/40	178	135	0.41	0.32
15-SJ	São José do Sul	3/30	2b + 2d	1/10	186	132	1.67	0.18
16-SN	Sede Nova	7/70	2d	2/20	181	124	1.49	0.56
17-SS	Salvador do Sul	7/70	2d	0/10	182	135	1.53	0.32
18-TA	Tupandi	1/10	2d	0/0	187	134	0.56	0.25
19-TB	Tupandi	8/80	2d	0/0	181	153	1.00	0.25
20-TU	Tucunduva	5/50	2d	1/10	184	123	0.91	0.80
21-UA	União da Serra	2/20	2d	1/10	178	127	0.51	0.72
22-UB	União da Serra	0/0	-	0/0	182	131	0.42	0.46
23-VC	Vespasiano Correa	4/40	2d	1/10	180	128	1.46	0.39
24-VF	Vila Flores	3/30	2b + 2d	1/10	185	134	0.75	0.56
25-VL	Vale Real	4/40	2d	0/0	187	130	0.69	0.62

**Table 2:** Main non-synonymous amino acid substitutions identified in the capsid protein-coding region of PCV2.

		Amino Acid Position															
PCV2 Sequence	Genotype	39	53	57	80	86	88	89	90	91	106	121	131	132	133	134	136
AF055392_Canada_1998	2a	R	F	V	V	T	K	I	S	I	W	T	T	K	A	T	L
KC835190_Brazil_2003	2b	.	.	I	L	S	P	R	.	V	.	S	.	.	.	.	.
EU148504_Denmark_1987	2c	.	.	.	L	S	P	L	T	V	F	.	.	.	.	.	.
PP230488_Brazil_2017	2d	.	I	.	L	S	P	L	T	V	.	.	.	.	.	N	.
EF524532_China_2006	2e	.	.	.	L	.	.	.	.	.	.	S	P	.	S	.	.
MF278779_China_1999	2f	.	.	.	L	S	P	L	T	V	.	.	.	.	.	.	.
JX099786_VietNam_2008	2g	.	.	.	L	S	P	L	T	V	.	.	.	.	.	.	.
JX506730_VietNan_2004	2h	.	.	.	L	S	P	L	T	V	.	.	P	.	.	.	.
This study (*n* = 1) Brazil_2025 ^1^	2b	.	.	I	L	S	P	R	.	V	.	S	.	.	.	.	.
This study (*n* = 5) Brazil_2024/5 ^2^	2b	.	.	I	L	S	P	R	.	V	.	S	.	.	.	P	.
This study (*n* = 5) Brazil_2025 ^3^	2d	.	I	.	L	S	P	L	T	V	.	.	.	.	H	D	M
This study (*n* = 1) Brazil_2025 ^4^	2d	-	-	-	L	S	P	L	T	V	.	.	.	.	H	D	M
This study (*n* = 1) Brazil_2025 ^5^	2d	.	I	.	L	S	P	L	T	V	.	.	.	R	H	D	M
This study (*n* = 17) Brazil_2025 ^6^	2d	K	I	.	L	S	P	L	T	V	.	.	.	.	H	D	M
This study (*n* = 17) Brazil_2024/5 ^7^	2d	.	I	.	L	S	P	L	T	V	.	.	.	.	H	D	M
This study (*n* = 1) Brazil_2025 ^8^	2d	.	I	.	L	S	P	L	T	V	.	.	.	.	.	N	.

^1^ VF05; ^2^ NB02, SJ04, AM07, PL08, and SJ02; ^3^ SS03, SS04, TB03, ER03, and ER09; ^4^ SN10; ^5^ GU10; ^6^ PL02, NB03, FV09, PL09, UA01, LA02, LA09, AM01, NB08, RE07, VC10, AM02, RE08, RS01, UA05, VF07, and VF08; ^7^ PA06, CO05, CO04, SJ05, SN03, PA04, CR04, CR01, TU08, TU09, HA05, FV01, HA07, VC07, GU09, VL06, and VL09; ^8^ TA04. Amino acid classification according to physicochemical properties: nonpolar/hydrophobic amino acids are shown in magenta; aromatic amino acids in purple; polar/hydrophilic, uncharged amino acids in green; polar/hydrophilic, positively charged (basic) amino acids in yellow; and polar/hydrophilic, negatively charged (acidic) amino acids in blue. The alignment includes representative sequences of each PCV2 genotype and sequences generated in the present study. Sequences from the present study showing 100% amino acid similarity at the positions presented were grouped and are represented by the corresponding sample number (n) below the table. Dots (.) indicate amino acids identical to the corresponding residues in the reference sequence AF055392_2a_Canada_1998, whereas dashes (–) indicate amino acid gaps. The complete amino acid alignment is provided in Table S4.

**Table 3:** Spearman’s correlation (ρ) and significance level (*p*) among zootechnical parameters of the evaluated pig herds/batches.

Variables		Average Weight (kg)	Mortality (%)	Condemnation (%)
Average Weight (kg)	r (*p*)	-	−0.29 (0.156)	−0.59 (0.001)
Mortality (%)	r (*p*)	−0.29 (0.156)	-	0.22 (0.284)
Condemnation (%)	r (*p*)	−0.59 (0.001)	0.22 (0.284)	-

Values of *p* < 0.05 indicate statistically significant correlations.

## Data Availability

The nucleotide sequences generated in this study have been deposited in GenBank under accession numbers [QB037558-QB037605].

## Supplementary Materials

The following supporting information can be downloaded at https://www.mdpi.com/article/10.3390/ani16213526/s1, Table S1: Metadata for the 48 PCV2 sequences generated in this study. Table S2: Reference sequences of porcine circovirus type 2 (PCV2), representing different genotypes, and the porcine circovirus type 1 (PCV1) outgroup used in the phylogenetic analysis. Table S3: Raw data of the qPCR for PCV2 and PCV3. Table S4: Amino acid alignment of the PCV2 capsid protein (Cap/ORF2), including 20 previously sequenced data and 48 new sequences generated in this study. Dots (.) indicate amino acids identical to those of the reference sequence (AF055392), while dashes (–) indicate gaps in the amino acid sequence. Table S5: Detection and frequency of PCV2/PCV3 and productive performance parameters of pigs in the 25 swine herds/batches/supplementary table. Figure S1. Maximum-likelihood phylogenetic tree based on the complete PCV2 cap gene nucleotide sequences/rectangular phylogram.
